# Use of Ketamine in Patients with Multifactorial Neuropathic Pain: A Systematic Review and Meta-Analysis

**DOI:** 10.3390/ph17091165

**Published:** 2024-09-03

**Authors:** Alejandro Bruna-Mejias, Vicente Baeza, Javiera Gamboa, Belen Baez Flores, Jessica San Martin, Constanza Astorga, Javiera Leyton, Pablo Nova-Baeza, Mathias Orellana-Donoso, Alejandra Suazo-Santibañez, Alvaro Becerra-Farfán, Gustavo Oyanedel-Amaro, Juan Jose Valenzuela-Fuenzalida

**Affiliations:** 1 Departamento de Ciencias y Geografía, Facultad de Ciencias Naturales y Exactas, Universidad de Playa Ancha, Valparaíso 2360072, Chile; alejandro.bruna@upla.cl; 2 Departamento de Morfología, Facultad de Medicina, Universidad Andres Bello, Santiago 8420524, Chile; vicentebaeza2000@gmail.com (V.B.); javigamboaa@gmail.com (J.G.); belenbaezflores@gmail.com (B.B.F.); jessicasanmartinp@gmail.com (J.S.M.); javiera.leyton.si@gmail.com (J.L.); pablo.nova@usach.cl (P.N.-B.); 3 Faculty of Medicine and Science, Universidad San Sebastián, Santiago 8420524, Chile; constanzaastorga21@gmail.com (C.A.); mathor94@gmail.com (M.O.-D.); 4 Escuela de Medicina, Universidad Finis Terrae, Santiago 7501015, Chile; 5 Faculty of Health and Social Sciences, Universidad de las Américas, Santiago 8370040, Chile; alej.suazo@gmail.com; 6 Escuela de Fonoaudiología & Departamento de Ciencias Química y Biológicas, Facultad de Ciencias de la Salud, Universidad Bernardo O’Higgins, Santiago 8320000, Chile; alvaro.becerra@ubo.cl; 7 Facultad de Ciencias de la Salud, Universidad Autónoma de Chile, Santiago 8910060, Chile; g.oyanedelamaro@gmail.com

**Keywords:** ketamine, pharmacology, neuropathic pain, polyneuropathy

## Abstract

Neuropathic pain (NP) is a heterogeneous group of conditions characterized by the experience of a number of sensory disturbances including pain, burning sensations, paroxysms of stabbing pain, dysesthesias, allodynia, and hyperalgesia. The above-mentioned sensations may occur in a specific dermatome area or other delimited region of the body. The objective of this review was to analyze the evidence for ketamine in multifactorial neuropathic pain. The research group systematically searched the databases MEDLINE (via PubMed), EMBASE, SCOPUS, the Cochrane Central Register of Controlled Trials, the Cumulative Index to Nursing and Allied Health Literature (Cinahl), and the Web of Science. The findings of this review show that different forms of low doses of ketamine (LDK) do not present statistically significant changes for any of the scales included. In this study, the total symptom score [standardized mean difference (SMD) = −3.59, confidence interval (CI) = −4.16 to −3.02, and *p* < 0.00001], neuropathy impairment score (SMD = −1.42, CI = −3.68 to 0.84, and *p* = 0.22), and neuropathy symptom checklist (SMD = −0.09, CI = −0.15 to −0.02, and *p* = 0.01) were taken into account. For finality compared to the use of a placebo, the findings suggest that LDK does not exhibit significant differences in terms of pain reduction and functionality. Moreover, no specific dosages are identified to support the use of LDK in the reduction in NP.

## 1. Introduction

Neuropathic pain (NP) constitutes a heterogeneous group of conditions characterized by the experience of a varied number of sensory alterations that include pain, burning sensations, paroxysms of stabbing pain, dysesthesias, allodynia, hyperalgesia, and hyperpathia. The mentioned sensations can occur in a dermatomal area or another delimited region. It is understood that NP involves neuronal responses where both peripheral and central pain signaling contribute to the generation of spontaneous pain and evoked aspects of pain, encompassing allodynia [1,2]. Several studies report that out of a total of 243 cancer patients, 77.78% (189 individuals) experience symptoms like NP or neuropathic diseases, secondary to cancer or its treatment, such as chemotherapy [1,3,4].

The relationship between NP and neuropathy was studied by Hannon in 2023 in a group of 14 patients, aged 35 to 75 years, with a withdrawal from the study due to chest pain. It was found that the prevalence of NP associated with different pathologies, in various anatomical areas, was 46.2% in the feet of the participants, 46.2% radiating to the legs, and 7.8% only in the hands. The pain was described as a ‘burning’ sensation (10/13 patients = 76.7%), tingling (5/13 = 38.5%), pain (3/13 = 23.1%), sharp pain (3/13 = 23.1%), and stinging discomfort (1/13 = 7.7%).

Ketamine is a medication used as an adjuvant analgesic for reducing postoperative pain. Its mechanism of action involves being a non-competitive antagonist of the *N*-methyl-D-aspartate receptor, suspending cholinergic transmission, and inhibiting the reuptake of norepinephrine and 5-hydroxytryptamine [5,6]. Inhibiting these receptors can prevent central sensitization and the activation of responses to peripheral painful stimuli. This prevents intracellular calcium entry, resulting in the decline of the central sensitization cascade and hyperexcitability, leading to reduced intensity and duration of postoperative pain.

In 2011, Niesters [7] reported that for a 70 kg patient, ketamine dosing began at 5 mg/h with a maximum infusion rate of 30 mg/h, which could be increased by 2.5 mg/h if pain relief was insufficient. On the other hand [8], it was analyzed that ketamine can be administered in 50 mg/5 mL vials intravenously with an electric syringe at a dose of 0.5 mg/kg diluted in 45 mL of physiological saline (0.9% NaCl) following pain clinic procedures.

Studies conducted by various authors [1,9,10] conclude that the most common adverse events of administering ketamine include anorexia, nausea, vomiting, drowsiness, dizziness, hallucinations, and tinnitus. Research by Monks in 2022 [11], reported that intravenous ketamine administration leads to many intolerable side effects; however, a safer alternative is topical administration.

Lynch’s 2005 study [2], involving 20 patients with neuropathic pain, exhibited symptoms such as dynamic tactile allodynia, pinprick hyperalgesia, or a combination of hyperalgesia, hyperesthesia, and allodynia in the affected area, assessed on the NRS-PI. For this, topical 1% ketamine was used, resulting in a 16% reduction in pain scores greater than or equal to the NRS-PI, and a 50% or greater reduction in pain scores was observed in the 10% of subjects.

Finally, a report by Song in 2018 [12] demonstrates that ketamine has other benefits in addition to reducing neuropathic cancer pain. Its topical form is used for the treatment of postherpetic neuralgia. Additionally, the administration of this drug aided in postoperative pain in total knee arthroplasty and increased knee flexion in these patients. The study by Timm [13] showed that ketamine can reduce morphine (opioid) consumption/administration after thoracotomy. However, it is unable to reduce persistent postsurgical pain. Additionally, Chumbley [14] demonstrated that this drug helps reduce continuous pain over spontaneous and mechanical evoked pain in patients suffering from nerve injury.

### Objective

The objective of this study is to demonstrate the benefits of using ketamine versus other therapeutic modalities in the treatment of patients with neuropathic pain of multifactorial origin.

## 2. Methods

### 2.1. Literature Search

This systematic review was conducted following the Preferred Reporting Items for Systematic Reviews and Meta-Analyses guidelines [15]. This review has been registered on PROSPERO with the following ID: CRD42024497118. The research team systematically searched electronic databases for the literature search, including MEDLINE (via PubMed), EMBASE, SCOPUS, the Cochrane Central Register of Controlled Trials, the Cumulative Index to Nursing and Allied Health Literature, and the Web of Science databases, covering records from the earliest time to January 2024. Randomized or controlled clinical trials that have been published in English or Spanish were included. The following keywords were used in different combinations: “ketamine”; “pharmacology”; “polineuropathy”; and “neuropathic pain”. The search strategies for each database are available in the supplemental content (see Supplementary Table S1). Two authors (VB and JJ-V) independently screened the titles and abstracts of the references retrieved from the searches. We obtained the full text for references that either author considered to be potentially relevant. We involved a third reviewer (PN-B) if a consensus could not be reached.

The inclusion criteria for the studies in this review were as follows: patients with NP, patients who were administered LDK, reports of pain, disability, and/or functionality, and studies that were randomized clinical trials and experimental studies. Studies were excluded if they were letters, case reports/series, reviews, or non-human trials, as well as studies that enrolled patients with other diseases or administered other therapies in addition to LDK or had no control group.

### 2.2. Data Extraction and Quality Assessment

Two authors (JG-A and JL) independently extracted relevant data for each trial. The following data were extracted from the original reports: (i) authors and year of publication, (ii) DM of study and the total number of participants, (iii) outcome, (iv) statistical values and main results, (v) geographical region, (vi) sex distribution, and (vii) doses of intervention and type of administration. The methodological quality of the included studies was evaluated by the Cochrane RoB2 tool [16]. This tool assesses the RoB2 across seven domains: generation of a random sequence, concealment of the randomization sequence, blinding of participants and treatments, blinding of the evaluation of the results, incomplete results, selective reporting of results, and other sources of bias. Each domain could be considered as having “low”, “unclear”, or “high” RoB2. Disagreements were resolved by discussion or determined by a third reviewer (JJV-F) if a consensus could not be reached. The agreement rate between the reviewers was calculated using kappa statistics, resulting in a substantial agreement with a value of 0.72.

### 2.3. Data Synthesis and Analysis

For the assessment of NP, two scales were used: the numeric rating scale (NRS) and visual analog scale (VAS) for meta-analysis. These scales were analyzed as continuous outcomes. The effect size was calculated as the standard mean difference (SMD). The SMD score was calculated using Cohen’s d as the effect size statistic, categorizing the effect sizes as trivial (<0.2), small (0.2–0.5), medium (0.6–0.8), or large (>0.8). Additionally, depending on the heterogeneity of the data, the Hartung–Knapp–Sidik–Jonkman random effect or Mantel–Haenszel fixed effect methods were used to quantify the pooled effect size of the studies included. We presented the effect sizes as SMD, with their respective 95% confidence intervals (CIs) in the range between 2 and −2. The heterogeneity of results across studies was evaluated using the I2 statistic, which considers 0–40% as “may not be important”, 30–60% as “moderate”, 50–90% as “substantial”, and 75–100% as “considerable” heterogeneity. Furthermore, we conducted a visual inspection to detect overlapping CIs in the forest plots as well as the corresponding *p*-values. The meta-analysis was performed using RevMan 5.4 [16].

### 2.4. Rating the Quality of Evidence

The synthesis and quality of evidence for each outcome were assessed using the Grading of Recommendation, Assessment, Development, and Evaluation (GRADE). The quality of the evidence was classified into four categories: high, moderate, low, and very low [17]. We used the GRADE profiler to import the data from RevMan 5.4 to create a ‘summary of findings’ table, which can be found in Supplementary Table S3.

## 3. Results

### 3.1. Study Selection

**Figure 1:**
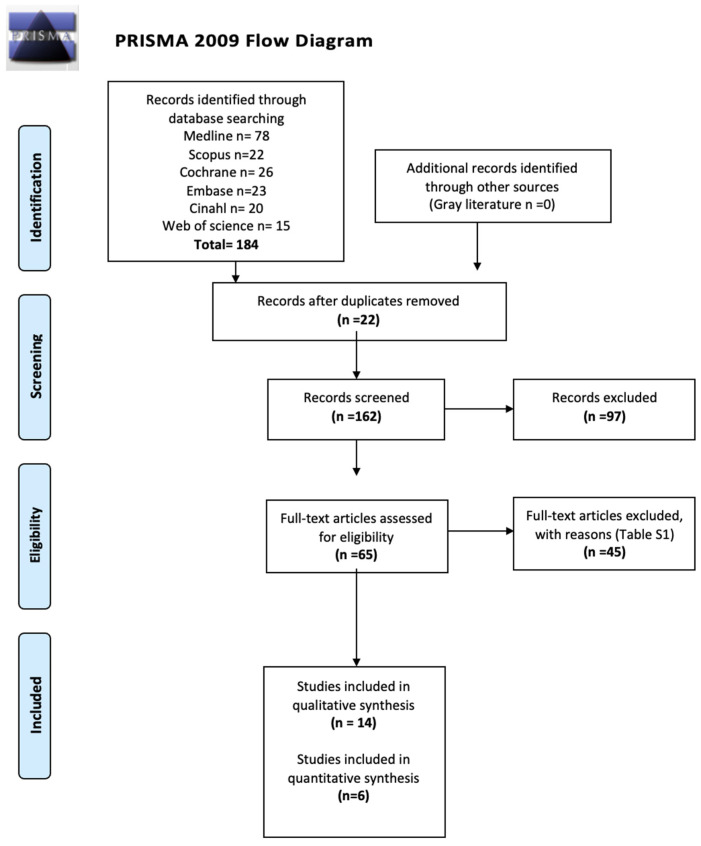
Flow diagram showing the study selection process based on the suggested format of Preferred Reporting Items for Systematic Reviews and Meta-Analyses guidelines.

Through electronic searches, a total of 184 studies were found (Figure 1). Ultimately, six trials met the eligibility criteria and were included in this systematic review and meta-analysis [18,19,20,21,22,23]. The kappa agreement rate between reviewers was 0.77. The excluded studies and the reasons for their exclusion are available in Supplementary Table S2 of the Supplemental Materials.

### 3.2. Descriptive Analysis of Studies Not Included in the Meta-Analysis

Within the included studies, 14 of them could not be pooled for meta-analysis [3,6,10,24,25,26,27,28,29,30,31,32,33,34]. The most frequently assessed scale is the NRS (numerical rating scale) in Yazigi et al., 2012 [34] (France), then ketamine (dose and administration) in Sigtermans et al., 2009 [33] (the Netherlands) and in Yazigi et al., 2012 [34] (France), and finally VAS (visual analog scale) in Sigtermans et al., 2009 [33] (the Netherlands). On the other hand, the studies of Yazigi et al., 2012 [34], France, and Sigtermans et al., 2009 [33], the Netherlands, showed statistically significant differences in some measurement scales. In relation to these escalations, the use of ketamine could have an effect that indicates that pain reduction is statistically significant, which supports the use of ketamine to manage pain in these patients. The studies mainly focus on non-functional types of pain, such as neuropathic, nociceptive, and chronic pain. In addition to treating patients with persistent and chronic pain, such as CRPS, post-spinal cord injury pain, phantom limb pain, cancer pain, and chronic postsurgical pain (Table 1).

### 3.3. Study Included Meta-Analysis Characteristics

A summary of the included studies is presented in Table 2. The overall population included 628 patients (315 in the KLD group and 313 in the placebo group). The mean age in the KLD group was 52.9 years (±2.1), the mean age in the placebo group was 53.4 years (±3.1), and the mean follow-up duration was 31 days (ranging from 1 to 84).

### 3.4. Risk of Bias Assessment in Individual Studies

**Figure 2:**
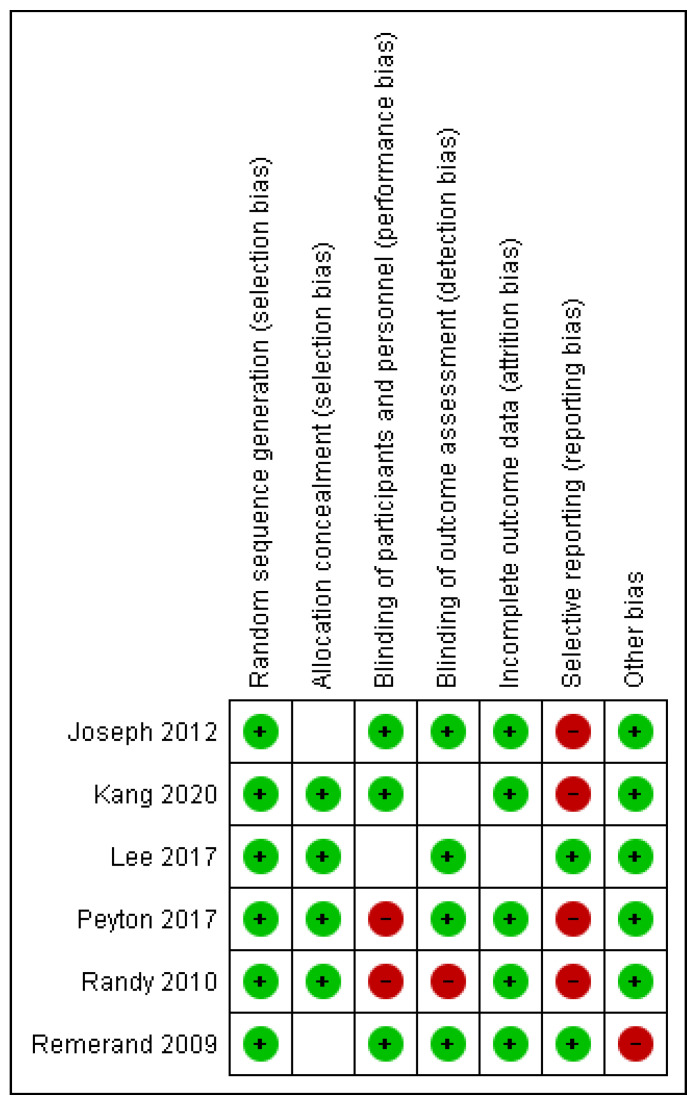
Risk of bias summary: review authors’ judgments about each risk of bias item for each included study [18,19,20,21,22,23].

Here, we present an assessment of risk of bias in individual studies. The evaluation of RoB2 is presented in Figure 2. In the random sequence generation, 100% of the studies were classified as “low risk” [18,19,20,21,22,23]. In allocation concealment, 66.66% were classified as “low risk” of bias [19,20,21,22], while 33.33% presented an “unclear risk” [18,23]. For blinding of participants and personnel, 50% of trials were rated “low risk” of bias [18,19,23], while 33.33% received a “high risk” rating [21,22] and 16.66% received a “unclear risk” rating [20]. For the blinding of outcome assessments, 66.66% of trials were rated “low risk” [18,20,21,23] and 16.66% “unclear risk” [19], while 16.66% received a “high risk” [22]. For incomplete outcome data, 83,66% received “low risk” [18,19,21,22,23], while 16.66% received “unclear risk” [20]. Finally, for the selection of the reported results, 33.33% of the trials were rated as “low risk” [20,23], while 66.66% received “high risk” [18,19,21,22].

### 3.5. Synthesis of Results

#### 3.5.1. Scales for Evaluation

Regarding the studies that showed some homogeneity in treatment and evaluation, six studies were included in this meta-analysis. We would like to highlight that these six studies were only included in the quantitative analysis since here the results of the outcomes between groups are shown and we can make comparisons between studies [18,19,20,21,22,23]. The evaluation scales used in these studies were NRS and VAS. The administration and dosage of LDK were performed orally and intravenously (IV), with doses of 0.5 mg/day. The results of each evaluation scale are detailed below.

##### NRS LDK First Month

**Figure 3:**
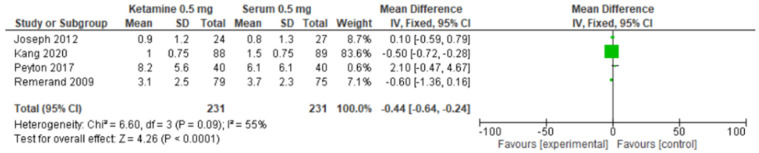
Forest plot of comparisons of the TSS standardized mean difference (SMD) between LDK600 EV and the placebo [18,19,20,21].

Four studies [18,19,20,21] provided data used to perform a meta-analysis to assess associated symptoms in patients with NP using the NRS scale. These studies showed a significant difference in the pooled SMD estimate between LDK 0.5 mg versus the placebo (serum 0.5 mg) (SMD = −0.44, CI = −0.64 to −0.24, and *p* < 0.001), with a substantial heterogeneity (I2 = 55% and *p* = 0.09) [18,19,20,21]. These results are presented in Figure 3. The quality of evidence, based on the GRADE rating, was determined to be low.

##### NRS LDK Third Month

**Figure 4:**
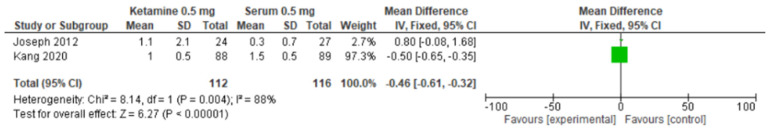
Forest plot of comparisons of the TSS standardized mean difference (SMD) between oral LDK600 and the placebo [18,19].

Two studies [18,19] provided data used to perform a meta-analysis to assess associated symptoms in patients with NP using the NRS scale. These studies showed no significant difference in the pooled SMD estimate between LDK 0.5 mg versus the placebo (serum 0.5 mg) (SMD = −0.46, CI = −0.61 to −0.32, and *p* < 0.001) and had substantial heterogeneity (I2 = 88% and *p* = 0.004) [18,19]. These results are presented in Figure 4. The quality of evidence, based on the GRADE rating, was determined to be very low.

##### VAS LDK First Month

**Figure 5:**
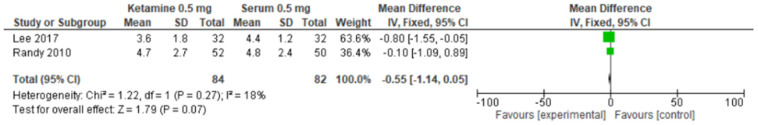
Forest plot of comparisons of the TSS standardized mean difference (SMD) between oral LDK1800 and the placebo [22,23].

Two studies [22,23] included data used to perform a meta-analysis to assess associated symptoms in patients with NP and using the VAS scale. These studies showed no significant difference in the pooled SMD estimate between LDK 0.5 mg versus the placebo (serum 0.5 mg) (SMD = −0.55, CI = −1.14 to −0.05, and *p* = 0.07) and had a substantial heterogeneity (I2 = 18% and *p* = 0.27) [22,23]. These results are presented in Figure 5. There was a low quality of evidence according to the GRADE rating.

## 4. Discussion

The objective of this systematic review and meta-analysis was to determine the clinical efficacy of the use of ketamine in patients with multifactorial NP. The main findings of this study, in different types of follow-up and scales, were that LDK showed a reduction in pain within the first month, as evaluated by the NRS, in comparison to the placebo. However, it does not have a significant benefit after three months or within the first month when measured with VAS, in terms of the reduction in NP symptoms, in comparison to the placebo.

To perform comparisons with previous meta-analyses or systematic reviews investigating the effect of ketamine on NP, we found two articles that align with the clinical and pharmacological parameters mentioned in this review. For Michelet’s study in 2018 [35], the results suggest moderate evidence supporting the efficacy of ketamine in chronic pain. The effect of ketamine in patients with PN, whether due to injury, surgery, or limb amputation, among others, has been studied in various conditions and in complex regional pain syndrome (CRPS), which considers neuropathic pain, chronic pain postsurgical and other severe pain conditions, demonstrating that ketamine can provide considerable relief from chronic pain in the short term; however, research on its long-term effects is less conclusive [35]

Additional studies are required to conclude on the effect of ketamine on chronic pain and to determine optimal administration regimens for this condition. Unlike the aforementioned study, our research presented a different outcome assessment, focusing on NP rather than chronic pain, showing that LDK yields positive results in NP management in the first month, distinguishing it from Michelet’s study in 2018. Regarding Zhao’s study in 2018 [36], the main findings indicate that ketamine infusion can provide short-term pain relief for complex regional pain syndrome, lasting less than three months. However, due to the high study heterogeneity and publication bias, additional randomized trials and standardized multicenter studies are needed to confirm this conclusion. Additionally, further studies are needed to support the efficacy of ketamine in the treatment of pain in complex regional pain syndrome. Our review reported a shorter efficacy duration of one month, and the outcome assessment differed as we focused on NP rather than complex regional pain syndrome. Additionally, the included studies did not report the LDK dosage. Regarding the dosage of LDK used, none of the aforementioned studies reported it [35,36].

In an analysis of 15 studies on CRPS, ketamine showed a notable decrease in pain scores. The immediate pain relief rate was 69%, and the 1–3-month pain relief rate was 58%. Reported adverse effects included anxiety, dysphoria, nightmares, hallucinations, insomnia, euphoria, agitation, blurred vision, and sedation. Additionally, elevations in liver enzymes were reported in two studies, suggesting that ketamine may induce hepatotoxicity through mitochondrial impairment. This patient’s liver enzyme levels did not return to normal until 2 months after the ketamine infusion was stopped [35].

In another study, ketamine was shown to have a significant reduction in pain scores compared to placebo in patients with chronic pain. The meta-analysis revealed a short-term analgesic effect up to 2 weeks after infusion. In the studies reviewed, doses ranged from 0.22 mg/kg to 0.6 mg/kg, and the duration of administration ranged from 30 min to 5 h, administered over several consecutive days. The types of pain included in these studies were mostly neuropathic, characterized by allodynia (pain due to stimuli that are not normally painful) and hyperalgesia (exaggerated response to painful stimuli); among them, severe post-spinal cord injury, phantom limb pain, opioid-refractory cancer pain, and fibromyalgia stand out [37].

While there is a limited variety in meta-analyses examining the use of LDK in patients with NP associated with other pathologies, it is worth mentioning that ketamine has been more extensively studied in pre- and postsurgical pain. Five systematic reviews and meta-analyses [38,39,40,41,42] have reported its benefits in reducing pre- and postsurgery pain, acute pain, especially in emergency services, and to a lesser extent, nausea reduction. An additional study on the use of perioperative ketamine showed that, compared with placebo, ketamine may not produce significant differences in the number of patients with chronic postsurgical pain after 6 months. Despite this, it can reduce the incidence of chronic postsurgical neuropathic pain after 3 months. The most notable adverse effects in this study group were an increased risk of nystagmus and postoperative visual disturbances. The doses used in these studies were between 0.2 mg/kg and 0.5 mg/kg, with infusions of 0.06 mg/kg/h to 0.18 mg/kg/h, administered over 24 to 48 h [43].

This underscores ketamine’s diverse applications, extensively documented in the literature, but this is the first meta-analysis associating it with NP.

The effects of ketamine are still under study; however, it is recognized as a general anesthetic and has been widely used in the treatment of refractory depression. Among its known mechanisms, ketamine antagonizes the NMDA receptor, leading to effects on synaptic plasticity and neuronal communication. It also increases the release of glutamate in specific brain areas, modulating the activity of monoamine receptors and contributing to its antidepressant effect. Additionally, ketamine interacts with opioid receptors, producing an analgesic effect. Studies have demonstrated these effects at a low dose of 0.1–0.3 mg/kg/h. In this review, we found evidence suggesting that even after treatment cessation, the beneficial effects persist or are maintained in chronic pain for at least three months. Further research is needed to comprehensively understand the long-term effects of ketamine in chronic pain management.

Based on the evidence we analyzed, we observed consistency in the dosage and administration methods of LDK among patients experiencing neuropathic pain (NP) linked to various conditions. The only variations in the studies suitable for meta-analysis were the outcome measures (NRS and VAS) and the follow-up periods of one and three months. In patients with NP stemming from both central and peripheral mechanisms, these factors significantly contribute to the onset of symptoms, leading to the exploration of various treatment options. While there is a wide range of pharmacological treatments available, many suggested medications lack substantial evidence supporting their efficacy. The clinical implications of our findings are constrained by the quality and quantity of the existing evidence. The use of different evaluation scales and follow-up durations resulted in considerable heterogeneity among the samples, preventing direct comparisons and necessitating the exclusion of some studies from the analysis. Consequently, we cannot make definitive clinical recommendations regarding the use of LDK for patients with NP, diabetes mellitus (DM), or other conditions. Although LDK may help alleviate pain, enhance functionality, or decrease disability, there is no agreement on the appropriate dosage, administration routes, or treatment duration. Additionally, research indicates that discontinuing LDK indefinitely or permanently might lead to adverse effects for patients. One proposed pharmacological option is LDK, which has shown some short-term benefits for symptomatic diabetic polyneuropathy. Studies have reported a noticeable reduction in symptoms after one month of continuous use; however, the analgesic effect tends to wane after five weeks. A recommended intravenous dose of LDK is 0.5 mg/kg, which has been found to improve pain management and is generally well-tolerated. The most common side effects reported include nausea, vomiting, and dizziness. Our findings indicate that LDK is effective in reducing NP during the first month, as measured by the NRS, although no significant differences were noted when assessed using the VAS. This discrepancy, despite identical administration times and dosages, may stem from the differences in assessment scales utilized across studies. If any studies using the NRS scale reported statistically significant results with greater weight, it could have skewed the forest plot of NRS at the one-month follow-up to show favorable results for LDK. A closer examination revealed that Peyton’s 2017 study exhibited a substantial change from its baseline, potentially influencing the pooled data to yield statistically significant outcomes. Notably, Peyton’s study did not assess VAS during the first month. In conclusion, while ketamine has demonstrated effectiveness for short-term pain relief in various chronic pain conditions or neuropathies, the evidence regarding its long-term effects remains unclear. Therefore, it is advised that broader experimental studies be conducted, as the variability in dosages and treatment durations highlights the need for further research to establish optimal treatment protocols and to better evaluate both the potential benefits and adverse effects associated with its use [20,35,44].

## 5. Limitations

This review has limitations. Firstly, the included studies may have publication bias: studies with different results that were in non-indexed literature in the selected databases may have been excluded. Secondly, there is a probability that a more sensitive and specific search regarding the topic to be studied was not carried out. Finally, personal preferences may have influenced the authors in the selection of articles. Other limitations include the change in objective after the registration of PROSPERO, the meta-analysis with only two studies giving weak conclusions.

## 6. Conclusions

The use of LDK compared to the use of placebos only showed significant differences within the first month of use in the reduction in NP, as measured by the NRS scale. In our study, the dose and the neurophysiological mechanism that can support the use of LDK for the reduction in NP were not found. It is important to note that based on the GRADE analysis, the evidence in favor of or against the use of LDK in patients with NP is low to moderate; therefore, additional high-quality studies with a large number of patients are needed. Finally, we believe that new primary studies supporting or refuting the use of LDK in the treatment of NP are necessary.

## Figures and Tables

**Table 1:** Characteristics included studies.

Reference	Country	Ketamine Group		Non-Ketamine Group		Results between Groups
		Patients	Intervention	Patients	Intervention	
Carver, et al., 2018 [24]	The United States of America	N = 45 Age = 49 Patients with multiples ribs fracture	Infusion of LDK (2.5 μg·kg^−1^·min^−1^) within 12 h of a patient’s arrival at the institution and were continued for a total of 48 h unless safety concerns prompted otherwise	N = 46 Age = 46 Patients with multiple rib fractures	A similar dose of placebo was administered	While no difference was noted in NPS or OME within the entire cohort at 12 h, 24 h, or 48 h, LDK significantly reduced OME utilization in severely injured patients (ISS, >15). NPS at 12–24 h: mean 5.9, SD 2.0, *p* = 0.36 NPS at 24–48 h: mean 5.7, SD 2.0, *p* = 0.77 OME at 12–24 h: mean 57.3, SD 57.1, *p* = 0.79 OME at 24–48 h, mean 99.6, SD 157.2, *p* = 0.63
Czarnetzki, et al., 2019 [25]	Switzerland	N = 80 Age = 67.0 Patients subjected to major lower back surgery	Intravenous ketamine 0.25 mg/kg preoperatively, followed by 0.25 mg/kg/h intraoperatively and 0.1 mg/kg/h from 1 h before the end of surgery until medical discharge	N = 80 Age = 66.0 Patients subjected to major lower back surgery	Similar volume of placebo (physiological saline), the dosing of which was based on ideal body weight.	DN4 score ≥ 4 at baseline *p* = 1, DN4 score ≥ 4 at 6 months *p* = 0.607, DN4 ≥ 4 at 12 months *p* = 0.319 Total score COMI at baseline *p* = 0.957; at 6 months *p* = 0.946; at 12 months *p* = 0.841 * MEAN AND SD *
Jafarinia, et al., 2016 [26]	Iran	N = 20 Age = 40.7 Patients with depression and chronic pain	50 mg ketamine (50 mg capsules) thrice daily for 6 weeks.	N = 20 Age = 38.95 Patients with depression and chronic pain	50 mg diclofenac (50 mg capsules) thrice daily for 6 weeks.	There was no significant difference between the mean VAS scores for ketamine and diclofenac arms at baseline, 3 weeks post-treatment, and at the study end point (72 ± 17.95 vs. 69.50 ± 18.77, *p*-value = 0.669, 55.70 ± 29.91 vs. 55.35 ± 30.07, *p*-value = 0.960, 55.25 ± 26.08 vs. 49.95 ± 30.58, *p*-value = 0.577; Analysis of GLM repeated- measure ANOVA confirmed the effect size of time × treatment was not significant throughout the trial period (F1.71, 64.84 = 0.289, *p*-value = 0.715). Mean (95%CI) difference in changes in the VAS score between ketamine and diclofenac intervention groups were not statistically different at week 3 or the study endpoint at week 6 (16.30 ± 17.86 vs. 14.25 ± 14.17; mean difference: ketamine−diclofenac (95% CI): 2.05 (−8.27 to 12.37); Cohen’s d: 0.13; *p*-value = 0.690 and 16.65 ± 22.67 vs. 19.55 ± 24.69; mean difference: ketamine−diclofenac (95% CI): −2.90 (−18.07 to 12.27); Cohen’s d: −0.12; *p*-value = 0.701
Jain, et al., 2022 [27]	India	N = 25 Age = 33.44 Patients undergoing elective laparoscopic cholecystectomy under general anesthesia of GRADE I or II with chronic pain	Ketamine (0.5 mg/kg) intravenous injection after LC	N = 25 Age = 37.64 Patients undergoing elective laparoscopic cholecystectomy under general anesthesia of GRADE I or II with chronic pain	Normal saline (2 mL) intravenous injection after LC	NRS at 1 h: mean 1.44; SD 0.77; *p* = 0.056 NRS at 2 h: mean 1.40; SD 0.76; *p* = 0.13 NRS at 4 h: mean 1.44; SD 0.82; *p* = 0.29 NRS at 6 h: mean 1.76; SD 1.09; *p* = 0.623 NRS at 8 h: mean 2.28; SD 1.34; *p* = 0.18 NRS at 12 h: mean 2.04; SD 1.17; *p* = 0.207 NRS at 24 h: mean 1.44; SD 0.82; *p* = 0.137
Lauretti, et al., 1999 [28]	Brazil	N = 12 Age = 56 Patients with terminal cancer suffering from chronic pain	Received 0.2 mg/kg epidural ketamine (2 mL)	N1 = 12 Age = 54 N2 = 12 Age = 50 N3 = 12 Age = 55 Patients with terminal cancer suffering from chronic pain	Group 1: received 2 mg of epidural morphine (2 mL). Group 2: received 2 mg of epidural morphine (2 mL). Group 3: received 500 mg epidural midazolam (2 mL).	VAS score: mean: 9; DS: 1: *p* = 0.222 morp
Lumanauw, et al., 2019 [29]	The United States of America	Group 1 N = 30 Age = 47.8 Group 2 N = 35 Age = 44.3 Patients with acute exacerbations of chronic pain	Group 1 = 0.5 mg/kg intravenous ketamine Group 2 = 0.25 mg/kg intravenous ketamine	n = 32 Age = 47.6 Patients with acute exacerbations of chronic pain	Similar volume of placebo (physiological saline), the dosing of which was based on ideal body weight	VAS at baseline: Pain group 1, mean 91.4, SD 8.5 Pain group 2, mean 93.2, SD 8.9 Pain group 3 (placebo), mean 91.2, SD 9.4 Both ketamine groups were superior to placebo, to successful improvement in their pain *p* = 0.001
Nielsen, et al., 2017 [30]	The United States of America	N = 74 Age = 57 Patients subjected to spinal fusion surgery	Intraoperative S-ketamine bolus 0.5 mg/kg, followed by an infusion 0.25 mg/kg*h	N = 73 Age = 55 Patients subjected to spinal fusion surgery	Isotonic sodium chloride, bolus, and infusion, the dosing of which was based on ideal body weight	Pain (VAS) at rest in the ketamine group, mean 46, SD 19 Placebo group, mean 48, SD 20 *p* = 0.62
Rakhman, et al., 2011 [31]	Israel	Group 1 (K1) N = 20 Age = 46 Group 2 (K2) N = 20 Age = 45 Group 3 (K3) N = 20 Age = 46 Patients undergoing tumor resection	Group 1 = Ketamine 25 mg at 4 h preoperatively Group 2 = ketamine 10 mg at 11 h preoperatively, and 25 mg at 4 h preoperatively Group 3 = ketamine 5 mg at 17 h preoperatively, 10 mg at 11 h preoperatively, and 25 mg at 4 h preoperatively	Group 1 (P1) N = 20 Age = 45 Group 2 (P2) N = 20 Age = 43 Group 3 (P3) N = 20 Age = 47 Patients undergoing tumor resection	Group 1 = 1 mL normal saline at 4 h preoperatively Group 2 = 1 mL normal saline at 11 and 4 h preoperatively Group 3 = 1 mL normal saline at 17, 11 and 4 h preoperatively	NRS (numerical rating scale), pain score K1 = NRS mean 5.4, SD = 2.06 K2 = NRS mean 6.41, SD = 0.95 K3 = NRS mean 6.28, SD = 1.1 P1 = NRS mean 5.0, SD = 1.71 P2 = NRS mean 4.69, SD = 1.69 P3 = NRS mean 5.16, SD = 1.75 Patients self-rated satisfaction scores were better in the K2 and K3 patients compared with their control counterparts (*p* < 0.005)
Rigo, et al., 2017 [32]	Brazil	Ketamine group N = 11 Age = 54 Patients with neuropathic chronic pain	Ketamine 30 mg oral, 3 times a day	Methadone group N = 13 Age = 52 Methadone + ketamine group N = 13 Age = 45 Patients with neuropathic chronic pain	Methadone 3 mg oral, 3 times a day Methadone + ketamine group = Methadone 3 mg, oral plus 30 mg of ketamine oral, 3 times a day	Visual analog scale (VAS) VAS after 90 days of treatment Ketamine group = mean 1.6, SD = 1.3 Methadone group = mean 1.3, SD = 1.0 Methadone + ketamine group= mean 2.2, SD = 1.1 *p* < 0.001
Sigtermans, et al., 2009 [33]	The Netherlands	N = 30 Age = 43.7 Patients with complex regional pain syndrome type 1	Ketamine 1.2 μg/kg per min, intravenous	N = 30 Age = 47.5 Patients with complex regional pain syndrome type 1	Similar volume of placebo (physiological saline), the dosing of which was based on ideal body weight	NRS (numerical rating scale), pain score at end of week one, ketamine group = mean 2.68, SD = 0.51 Placebo group = mean 5.35, SD = 0.48 *p* < 0.001
Yazigi, et al., 2012 [34]	Lebanon	N = 30 Age = 57.3 Patients subjected to a thoracotomy	Ketamine (0.1 mg/kg as a preincisional bolus followed by a continuous infusion of 0.05 mg/kg/h)	N = 30 Age = 56.9 Patients subjected to a thoracotomy	Similar volume of placebo (physiological saline), the dosing of which was based on ideal body weight	They were not significantly different between the two groups at any time point of the study, at rest (*p* = 0.75) or during coughing (*p* = 0.70)
Aveline, et al., 2014 [10]	France	N = 24 Age = 71 Patients with osteoarthritis scheduled for elective tricompartmental TKA performed	Ketamine 0.2 mL/kg bolus over 20 min started before surgical incision, followed by a continuous infusion of 120 mg/kg/h until the end of surgery and then 60 mg/kg/h until the second post- operative day	N nefopam = 22 Age = 71 N placebo = 23 Age = 71 Patients with osteoarthritis scheduled for elective tricompartmental TKA	Nefopam 0.2 mL/kg bolus over 20 min started before surgical incision, followed by a continuous infusion of 120 mg/kg/h until the end of surgery and then 60 mg/kg/h until the second postoperative day Infusion of isotonic saline	The RR of having CP during movement was not significantly decreased by ketamine and nefopam (ketamine vs. placebo: RR 0.48 [95% CI, 0.14- 1.69, *p* = 0.25]; nefopam vs. placebo: RR 0.52 [95% CI, 0.15–1.84, *p* = 0.31]). Ketamine and nefopam did not decrease the RR of having a DN4 score Z4 at M12 compared with placebo (RR 0.48 [95% CI, 0.1–2.37], *p* = 0.36 and RR 0.27 [95% CI, 0.03–2.16], *p* = 0.22, respectively). No difference was documented between ketamine and nefopam (RR 1.91; 95% CI, 0.19–19.52; *p* = 0.59).
Hardy, et al., 2012 [3]	Australia	N = 93 Age = 63.0 Patients with cancer pain	Subcutaneous infusion of ketamine at three doses levels (100, 300, or 500 mg)	N = 92 Age = 64.3 Patients with cancer pain	Similar volume of placebo (physiological saline), the dosing of which was based on ideal body weight	BPI pain score Ketamine group average mean 5.43, SD = 1.3 Placebo group average mean 5.21, SD = 1.4 The difference in absolute terms is small (0.71) and was not clinically significant because the difference was not ≥BPI units.
Hassan, et al., 2021 [6]	Egypt	N = 44 Age = 50.14 Patients undergoing cancer breast surgeries	Ketamine group (K) 0.5 mg/kg bolus, then 0.12 mg/kg/h infusion for the first 24 h postoperatively	N = 43 Age = 50.91 Patients undergoing cancer breast surgeries	Group KM: ketamine 0.5 mg/kg and mg sulfate 50 mg/kg, then ketamine 0.12 mg/kg/h and Mg sulfate 8 mg/kg/h infusions for the first 24 h postoperatively	NRS (numerical rating scale), pain score Group K in the first 24 h at Rest = mean 1, SD = X Movement = mean 3, SD = X Group KM in the first 24 h Rest = mean 1, SD = X Movement = mean 3, SD = X Rest *p* = 0.193 Movement *p* = 0.255

**Table 2:** Summary of the characteristics of the studies included in the meta-analysis.

Author	Country	Total N in Experimental Group	Characteristics and Doses in Experimental Group	Total N in Control Group	Characteristics and Doses in Control Group	Outcomes
Peyton, et al., 2017 [20]	The United States of America	N = 40 Age = 55.3 Patients with chronic pain after a thoracic or abdominal surgery	Ketamine 0.5 mg/kg preincision, 0.25 mg/kg/hour intraoperatively and 0.1 mg/kg/ hour for 24 h	N = 40 Age = 55.3 Patients with chronic pain after a thoracic or abdominal surgery	Similar volume of placebo (physiological saline), the dosing of which was based on ideal body weight.	NRS pain severity score (median [interquartile range, IQR]) for average pain in the previous 24 h among those patients reporting CPSP was 17.5/100 (IQR (0–40))
Remérand, et al., 2009 [21]	France	N = 79 Age = 64 Patients with chronic pain after total hip arthroplasty	Ketamine 0.5 mg/kg IV before incision and a 24 h infusion of ketamine 2 μg/kg per min	N = 75 Age = 65 Patients with chronic pain after total hip arthroplasty	Similar volume of placebo (physiological saline), the dosing of which was based on ideal body weight.	NRS (numerical rating scale), worst pain score, day 0 to day 3 pain NRS Ketamine = mean 41, SD = 28 Placebo = mean 45, SD = 35 Worst, day 4 to day 7 pain NRS Ketamine = mean 31, SD = 25 Placebo = mean 37, SD = 23
Joseph, et al., 2012 [18]	France	N = 24 Age = 60 Patients planned for an elective partial pneumonectomy (partial or total lobectomy involving one or more lobes, except total pneumonectomy) by posterolateral or lateral thoracotomy	Received a combination of the continuous i.v. infusion of ketamine for 48 h and patient-controlled thoracic epidural analgesia (PCEA) with ropivacaine 1.5 mg/mL during the thoracotomy postoperative period. An i.v. ketamine infusion was standardized as follows: 0.5 mg/kg of ketamine during anesthesia induction and an intraoperative continuous i.v. infusion of ketamine 3 μg kg^−1^ min^−1^ following by a postoperative infusion of ketamine 1.5 μg kg^−1^ min^−1^ during the postoperative 48 h, starting at the end of the surgery	N = 27 Age = 60 Patients planned for an elective partial pneumonectomy (partial or total lobectomy involving one or more lobes, except total pneumonectomy) by posterolateral or lateral thoracotomy	Were given a combination of continuous i.v. infusion of saline solution and PCEA with ropivacaine 1.5 mg/mL during the thoracotomy postoperative period. The saline solution was administered using the same protocol and the same duration	NRS (rest) first month in ketamine group: mean: 0.9; SD: 1.2; *p* = 0.827 NRS (rest) third month in ketamine group: mean: 1.1; SD: 2.1; *p* = 0.385 NRS (abduction) first month in ketamine group: mean: 1.2; SD: 1.5; *p* = 0.909 NRS (abduction) third month in ketamine group: mean: 1.3; SD: 2.5; *p* = 0.589
Kang, et al., 2020 [19]	South Korea	N = 88 Age = 49.7 Patients scheduled for elective unilateral breast cancer surgery	Infusion of 100 mg of ketamine (2 mg/mL) with 48 mL of 0.9% normal saline	N = 89 Age = 50.8 Patients scheduled for elective unilateral breast cancer surgery	Infusion of 50 mL of 0.9% normal saline	NRSr after 1 month: mean: 1.0; interquartile range: 0–3.0; *p* = 0.667 NRSr after 3 months: mean: 1.0; interquartile range: 0–2.0; *p* = 0.696 NRSr after 6 months: mean: 0; interquartile range: 0–2.0; *p* = 0.929 NRSd after 1 month: mean: 3.0; interquartile range: 1.0–4.0; *p* = 0.168 NRSd after 3 months: mean: 2.0; interquartile range: 0–3.0; *p* = 0.119 NRSd after 6 months: mean: 1.0; interquartile range: 0–3.0; *p* = 0.474 DN-4 after 1 month: mean: 5.0; *p* = 0115 DN-4 after 3 months: mean: 3.0; *p* = 0.720 DN4-4 after 6 months: mean: 1.0; *p* = 0.210
Randy, et al., 2010 [22]	The United States of America	N = 52 Age = 51.7 Patients with chronic back pain undergoing back surgery	Ketamine 0.5 mg/kg intravenous on induction of anesthesia, and a continuous infusion at 10 μg/kg per min on induction and terminated at wound closure.	N = 50 Age = 51.4 Patients with chronic back pain undergoing back surgery	Similar volume of placebo (physiological saline), the dosing of which was based on ideal body weight.	Visual analog scale (VAS) VAS 24 h Ketamine group = mean 4.7, SD = 2.7 Placebo group = mean 4.8, SD = 2.4 *p* = 0.902 VAS 48 h Ketamine group = mean 5.4, SD = 2.1 Placebo group = mean 5.3, SD = 2.2 *p* = 0.838 VAS 6 weeks Ketamine group = mean 3.1, SD = 2.4 Placebo group = mean 4.2, SD = 2.4 *p* = 0.026
Lee, et al., 2017 [23]	South Korea	N = 32 Age = 37 Patients scheduled for robotic thyroidectomy	Bolus dose of 0.15 mg/kg of racemic ketamine after anesthetic induction. Racemic ketamine was also infused continuously until the end of the surgery at a rate of 2 mg/kg/min	N = 32 Age = 38 Patients scheduled for robotic thyroidectomy	Similar volume of placebo (physiological saline), the dosing of which was based on ideal body weight.	There was a statistically significant difference in the VAS pain scores at rest and while coughing until 24 h postoperatively between the two groups (*p* = 0.028 and *p* = 0.039, respectively)

## Supplementary Materials

The following supporting information can be downloaded at: https://www.mdpi.com/article/10.3390/ph17091165/s1, Table S1: Search strategy in databases. Table S2: Excluded studies and reasons. Table S3: GRADE. Summary of Findings (SoF) and quality of evidence (GRADE) for Duloxetine in patients with neuropathic pain associated.

## Abbreviations

Neuropathic pain	NP
Low doses of ketamine	LDK
Intravenous	IV
Numeric pain score	NPS
Oral morphine equivalent	OME
Douleur neuropathique-4 items	DN4
Numeric rating scale	NRS
Visual analog scale	VAS
Brief pain inventory	BPI
Peripheral neuropathy	PN

## References

[1] Kannan T.R., Saxena A., Bhatnagar S., Barry A. (2002). Oral ketamine as an adjuvant to oral morphine for neuropathic pain in cancer patients. J. Pain Symptom Manag..

[2] Lynch M.E., Clark A.J., Sawynok J., Sullivan M.J.L. (2005). Topical 2% Amitriptyline and 1% Ketamine in Neuropathic Pain Syndromes: A Randomized, Double-blind, Placebo-controlled Trial. Anesthesiology.

[3] Hardy J., Quinn S., Fazekas B., Plummer J., Eckermann S., Agar M., Spruyt O., Rowett D., Currow D.C. (2012). Randomized, double-blind, placebo-controlled study to assess the efficacy and toxicity of subcutaneous ketamine in the management of cancer pain. J. Clin. Oncol..

[4] Fallon M.T., Wilcock A., Kelly C.A., Paul J., Lewsley L.-A., Norrie J., Laird B.J.A. (2018). Oral Ketamine vs Placebo in Patients with Cancer-Related Neuropathic Pain: A Randomized, Clinical Trial. JAMA Oncol..

[5] Hannon C.P., Fillingham Y.A., Gililland J.M., Sporer S.M., Casambre F.D., Verity T.J., Woznica A., Nelson N., Hamilton W.G., Della Valle C.J. (2022). A Systematic Review of the Efficacy and Safety of Ketamine in Total Joint Arthroplasty. J. Arthroplast..

[6] Hassan M.E., Mahran E. (2021). Effect of magnesium sulfate with ketamine infusions on intraoperative and postoperative analgesia in cancer breast surgeries: A randomized double-blind trial. Braz. J. Anesthesiol..

[7] Niesters M., Hoitsma E., Sarton E., Aarts L., Dahan A. (2011). Offset Analgesia in Neuropathic Pain Patients and Effect of Treatment with Morphine and Ketamine. Anesthesiology.

[8] Delage N., Morel V., Picard P., Marcaillou F., Pereira B., Pickering G. (2017). Effect of ketamine combined with magnesium sulfate in neuropathic pain patients (KETAPAIN): Study protocol for a randomized controlled trial. Trials.

[9] Kvarnström A., Karlsten R., Quiding H., Emanuelsson B., Gordh T. (2003). The effectiveness of intravenous ketamine and lidocaine on peripheral neuropathic pain. Acta Anaesthesiol. Scand..

[10] Aveline C., Le Roux A., Le Hetet H., Gautier J.F., Vautier P., Cognet F., Bonnet F. (2014). Pain and recovery after total knee arthroplasty: A 12-month follow-up after a prospective randomized study evaluating nefopam and ketamine for early rehabilitation. Clin. J. Pain.

[11] Monks D.T., Palanisamy A., Jaffer D., Singh P.M., Carter E., Lenze S. (2022). A randomized feasibility pilot-study of intravenous and subcutaneous administration of ketamine to prevent postpartum depression after planned cesarean delivery under neuraxial anesthesia. BMC Pregnancy Childbirth.

[12] Song D., He A., Xu R., Xiu X., Wei Y. (2018). Efficacy of Pain Relief in Different Postherpetic Neuralgia Therapies: A Network Me-ta-Analysis. Pain Physician.

[13] Timm C., Linstedt U., Weiss T., Zenz M., Maier C. (2008). Sympathomimetic effects of low-dose S(+)-ketamine: Effect of propofol dosage. Anaesthesist.

[14] Chumbley G.M., Thompson L., Swatman J.E., Urch C. (2019). Ketamine infusion for 96 hr after thoracotomy: Effects on acute and persistent pain. Eur. J. Pain.

[15] Page M.J., McKenzie J.E., Bossuyt P.M., Boutron I., Hoffmann T.C., Mulrow C.D., Shamseer L., Tetzlaff J.M., Akl E.A., Brennan S.E. (2021). PRISMA 2020 statement: An updated guideline for reporting systematic reviews. BMJ.

[16] Eldridge S., Campbell M.K., Campbell M.J. Revised Cochrane Risk of Bias Tool for Randomized Trials (RoB2). Additional Considerations for Cluster-Randomized Trials (RoB 2 CRT). https://www.riskofbias.info/welcome/rob-2-0-tool/rob-2-for-cluster-randomized-trials.

[17] Fritz C.O., Morris P.E., Richler J.J. (2012). Effect size estimates: Current use, calculations, and interpretation. J. Exp. Psychol. Gen..

[18] Joseph C., Gaillat F., Duponq R., Lieven R., Baumstarck K., Thomas P., Penot-Ragon C., Kerbaul F. (2012). Is there any benefit to adding intravenous ketamine to patient-controlled epidural analgesia after thoracic surgery? A randomized double-blind study. Eur. J. Cardio-Thorac. Surg..

[19] Kang C., Cho A.-R., Kim K.-H., Lee E.-A., Lee H.-J., Kwon J.-Y., Kim H., Kim E., Baik J.-S., Kim C. (2020). Effects of Intraoperative Low-Dose Ketamine on Persistent Postsurgical Pain after Breast Cancer Surgery: A Prospective, Randomized, Controlled, Double-Blind Study. Pain Physician.

[20] Peyton P.J., Wu C., Jacobson T., Hogg M., Zia F., Leslie K. (2017). The effect of a perioperative ketamine infusion on the incidence of chronic postsurgical pain—A pilot study. Anaesth. Intensiv. Care.

[21] Remérand F., Le Tendre C., Baud A., Couvret C., Pourrat X., Favard L., Laffon M., Fusciardi J. (2009). The Early and delayed analgesic effects of ketamine after total hip arthroplasty: A prospective, randomized, controlled, double-blind study. Anesth. Analg..

[22] Loftus R.W., Yeager M.P., Clark J.A., Brown J.R., Abdu W.A., Sengupta D.K., Beach M.L. (2010). Intraoperative ketamine reduces perioperative opiate consumption in opiate-dependent patients with chronic back pain undergoing back surgery. Anesthesiology.

[23] Lee J., Park H.-P., Jeong M.-H., Son J.-D., Kim H.-C. (2017). Efficacy of ketamine for postoperative pain following robotic thyroidectomy: A prospective randomised study. J. Int. Med Res..

[24] Carver T.W., Kugler N.W., Juul J., Peppard W.J., Drescher K.M., Somberg L.B., Szabo A., Yin Z., Paul J.S. (2019). Ketamine infusion for pain control in adult patients with multiple rib fractures: Results of a randomized control trial. J. Trauma Inj. Infect. Crit. Care.

[25] Czarnetzki C., Desmeules J., Tessitore E., Faundez A., Chabert J., Daali Y., Fournier R., Dupuis-Lozeron E., Cedraschi C., Tramèr M.R. (2020). Perioperative intravenous low-dose ketamine for neuropathic pain after major lower back surgery: A randomized, placebo-controlled study. Eur. J. Pain.

[26] Jafarinia M., Afarideh M., Tafakhori A., Arbabi M., Ghajar A., Noorbala A.A., Saravi M.A., Agah E., Akhondzadeh S. (2016). Efficacy and safety of oral ketamine versus diclofenac to alleviate mild to moderate depression in chronic pain patients: A double-blind, randomized, controlled trial. J. Affect. Disord..

[27] Jain S., Nazir N., Mustafi S. (2022). Preemptive low-dose intravenous ketamine in the management of acute and chronic postoperative pain following laparoscopic cholecystectomy: A prospective randomized control study. Med. Gas Res..

[28] Lauretti G.R., Gomes J.M., Reis M.P., Pereira N.L. (1999). Low doses of epidural ketamine or neostigmine, but not midazolam, improve morphine analgesia in epidural terminal cancer pain therapy. J. Clin. Anesth..

[29] Lumanauw D.D., Youn S., Horeczko T., Yadav K., Tanen D.A. (2019). Subdissociative-dose ketamine is effective for treating acute exacerbations of chronic Pain. Acad. Emerg. Med..

[30] Nielsen R.V., Fomsgaard J.S., Siegel H., Martusevicius R., Nikolajsen L., Dahl J.B., Mathiesen O. (2017). Intraoperative ketamine reduces immediate postoperative opioid consumption after spinal fusion surgery in chronic pain patients with opioid dependency: A randomized, blinded trial. Pain.

[31] Rakhman E., Shmain D., White I., Ekstein M.P., Kollender Y., Chazan S., Dadia S., Bickels J., Amar E., Weinbroum A.A. (2011). Repeated and escalating preoperative subanesthetic doses of ketamine for postoperative pain control in patients undergoing tumor resection: A randomized, placebo-controlled, double-blind trial. Clin. Ther..

[32] Rigo F.K., Trevisan G., Godoy M.C., Rossato M.F., Dalmolin G.D., A Silva M., Menezes M.S., Caumo W., Ferreira J. (2017). Management of Neuropathic Chronic Pain with Methadone Combined with Ketamine: A Randomized, Double Blind, Active-Controlled Clinical Trial. Pain Physician.

[33] Sigtermans M.J., van Hilten J.J., Bauer M.C., Arbous S.M., Marinus J., Sarton E.Y., Dahan A. (2009). Ketamine produces effective and long-term pain relief in patients with Complex Regional Pain Syndrome Type 1. Pain.

[34] Yazigi A., Abou-Zeid H., Srouji T., Madi-Jebara S., Haddad F., Jabbour K. (2012). The effect of low-dose intravenous ketamine on continuous intercostal analgesia following thoracotomy. Ann. Card. Anaesth..

[35] Michelet D., Brasher C., Horlin A., Bellon M., Julien-Marsollier F., Vacher T., Pontone S., Dahmani S. (2018). Ketamine for chronic non-cancer pain: A meta-analysis and trial sequential analysis of randomized controlled trials. Eur. J. Pain.

[36] Zhao J., Wang Y., Wang D. (2018). The Effect of Ketamine Infusion in the Treatment of Complex Regional Pain Syndrome: A Systemic Review and Meta-analysis. Curr. Pain Headache Rep..

[37] Orhurhu V., Orhurhu M.S., Bhatia A., Cohen S.P. (2019). Ketamine Infusions for Chronic Pain: A Systematic Review and Meta-analysis of Randomized Controlled Trials. Anesth. Analg..

[38] Włodarczyk A., Cubała W.J. (2020). Safety and Tolerability of Ketamine Use in Treatment-Resistant Bipolar Depression Patients with Regard to Central Nervous System Symptomatology: Literature Review and Analysis. Medicina.

[39] Murck H. (2013). Ketamine, magnesium and major depression—From pharmacology to pathophysiology and back. J. Psychiatr. Res..

[40] Brinck E.C., Tiippana E., Heesen M., Bell R.F., Straube S., Moore R.A., Kontinen V. (2018). Perioperative intravenous ketamine for acute postoperative pain in adults. Cochrane Database Syst. Rev..

[41] Balzer N., McLeod S.L., Walsh C., Grewal K. (2021). Low-dose Ketamine for Acute Pain Control in the Emergency Department: A Systematic Review and Meta-analysis. Acad. Emerg. Med..

[42] Zhou L., Yang H., Hai Y., Cheng Y. (2022). Perioperative Low-Dose Ketamine for Postoperative Pain Management in Spine Surgery: A Systematic Review and Meta-Analysis of Randomized Controlled Trials. Pain Res. Manag..

[43] Abouarab A.H., Brülle R., Aboukilila M.Y., Weibel S., Schnabel A. (2024). Efficacy and safety of perioperative ketamine for the prevention of chronic postsurgical pain: A meta-analysis. Pain Pract..

[44] Niciu M.J., Luckenbaugh D.A., Ionescu D.F., Richards E.M., Voort J.L.V., Ballard E.D., Brutsche N.E., Furey M.L., Zarate C.A. (2014). Ketamine’s antidepressant efficacy is extended for at least four weeks in subjects with a family history of an alcohol use disorder. Int. J. Neuropsychopharmacol..

