# Supplementary material for: Use of Ketamine in Patients with Multifactorial Neuropathic Pain: A Systematic Review and Meta-Analysis
Source: Pharmaceuticals (Basel). 2024 Sep 3;17(9):1165. doi: 10.3390/ph17091165 (PMC11435086; doi:10.3390/ph17091165)
Supplement: Supplementary file 1 [file pharmaceuticals-17-01165-s001.zip › pharmaceuticals-3156920 supplementary for tables.pdf]

pharmaceuticals

Table S1. Search strategy in databases.

Database	Search strategy	Results	
		29-07-24	
Medline	(("esketamine"[Supplementary Concept] OR "esketamine"[All Fields] OR "ketamine"[All Fields] OR "ketamine"[MeSH Terms] OR "ketamin"[All Fields] OR "ketamine s"[All Fields] OR "ketamines"[All Fields] OR (("esketamine"[Supplementary Concept] OR "esketamine"[All Fields] OR "ketamine"[All Fields] OR "ketamine"[MeSH Terms] OR "ketamin"[All Fields] OR "ketamine s"[All Fields] OR "ketamines"[All Fields]) AND "Low"[All Fields] AND "dose"[All Fields]) OR "LDK"[All Fields] OR (("esketamine"[Supplementary Concept] OR "esketamine"[All Fields] OR "ketamine"[All Fields] OR "ketamine"[MeSH Terms] OR "ketamin"[All Fields] OR "ketamine s"[All Fields] OR "ketamines"[All Fields]) AND ("pharmacology"[MeSH Terms] OR "pharmacology"[All Fields] OR "pharmacologies"[All Fields] OR "pharmacology"[MeSH Subheading]))) AND ("pain"[MeSH Terms] OR "pain"[All Fields])) OR ("neuralgia"[MeSH Terms] OR "neuralgia"[All Fields] OR ("neuropathic"[All Fields] AND "pain"[All Fields]) OR "neuropathic pain"[All Fields])	78	
Wos	Ketamine OR Ketamine Low dose OR LDK OR ketamine pharmacology AND pain OR neuropathic pain	15	
CINAHL	Ketamine OR Ketamine Low dose OR LDK OR ketamine pharmacology AND pain OR neuropathic pain	20	
SCOPUS	Ketamine OR Ketamine Low dose OR LDK OR ketamine pharmacology AND pain OR neuropathic pain	22	
Cochrane	Ketamine OR Ketamine Low dose OR LDK OR ketamine pharmacology AND pain OR neuropathic pain	26	
EMBASE	Ketamine OR Ketamine Low dose OR LDK OR ketamine pharmacology AND pain OR neuropathic pain	23	
	Total	184	

* All searches were carried out on July 29, 2024.

Table S2. Excluded studies and reasons.

	Reference	Reason
1	Bouwense SAW, Buscher HCJL, van Goor H, Wilder-Smith OHG. S-ketamine modulates hyperalgesia in patients with chronic pancreatitis pain. Reg Anesth Pain Med. 2011;36(3):303–7. http://dx.doi.org/10.1097/aap.0b013e3182177022	Same patients in different times
2	Carr DB, Goudas LC, Denman WT, Brookoff D, Staats PS, Brennen L, et al. Safety and efficacy of intranasal ketamine for the treatment of breakthrough pain in patients with chronic pain: a randomized, double-blind, placebo-controlled, crossover study. Pain . 2004;108(1–2):17–27. http://dx.doi.org/10.1016/j.pain.2003.07.001	No control group present for comparison.
3	Abram S. Chronic phantom limb pain: The effects of calcitonin, ketamine, and their combination on pain and sensory thresholds. Yearb Anesthesiol Pain Manag. 2009;2009:288–90. http://dx.doi.org/10.1016/s1073-5437(08)79031-4	No control group present for comparison.
4	Felsby S, Nielsen J, Arendt-Nielsen L, Jensen TS. NMDA receptor blockade in chronic neuropathic pain: a comparison of ketamine and magnesium chloride. Pain. 1996;64(2):283–91. http://dx.doi.org/10.1016/0304-3959(95)00113-1	No control group present for comparison.
5	Fernandes M, Schelotto M, Doldi PM, Milani G, Ariza Manzano AA, Perera Valdivia D, et al. Importance trial: a provisional study-design of a single-center, phase II, double-blinded, placebo-controlled, randomized, 4-week study to compare the efficacy and safety of intranasal esketamine in chronic opioid refractory pain. F1000Research . 2021;10:42. http://dx.doi.org/10.12688/f1000research.27809.1	Data isn't available
6	Furuhashi-Yonaha A, Iida H, Asano T, Takeda T, Dohi S. Short- and long-term efficacy of oral ketamine in eight chronic-pain patients. Can J Anaesth. 2002;49(8):886–7. http://dx.doi.org/10.1007/bf03017431	Same patients in different times
7	Lynch ME, Clark AJ, Sawynok J. A pilot study examining topical amitriptyline, ketamine, and a combination of both in the treatment of neuropathic pain. Clin J Pain. 2003;19(5):323–8. http://dx.doi.org/10.1097/00002508-200309000-00007	No control group present for comparison.
8	Jin L, Liang Y, Yu Y, Miao P, Huang Y, Xu L, et al. Evaluation of the effect of new multimodal analgesia regimen for cardiac surgery: A prospective, randomized controlled, single-center clinical study. Drug Des Devel Ther. 2023;17:1665–77. http://dx.doi.org/10.2147/DDDT.S406929	No control group present for comparison.
9	Khalili-Mahani N, Niesters M, van Osch MJ, Oitzl M, Veer I, de Rooij M, et al. Ketamine interactions with biomarkers of stress: a randomized placebo-controlled repeated measures resting-state fMRI and PCASL pilot study in healthy men. Neuroimage. 2015;108:396–409. http://dx.doi.org/10.1016/j.neuroimage.2014.12.050	No control group present for comparison.
10	Lemming D, Sörensen J, Graven-Nielsen T, Arendt-Nielsen L, Gerdle B. The responses to pharmacological challenges and experimental pain in patients with chronic whiplash-associated pain. Clin J Pain. 2005;21(5):412–21. http://dx.doi.org/10.1097/01.ajp.0000126155.82815.fc	No control group present for comparison.

11	Lemming D, Sörensen J, Graven-Nielsen T, Lauber R, Arendt-Nielsen L, Gerdle B. Managing chronic whiplash associated pain with a combination of low-dose opioid (remifentanil) and NMDA-antagonist (ketamine). Eur J Pain. 2007;11(7):719–32.http://dx.doi.org/10.1016/j.ejpain.2006.11.002	No control group present for comparison.
12	Niesters M, Aarts L, Sarton E, Dahan A. Influence of ketamine and morphine on descending pain modulation in chronic pain patients: a randomized placebo-controlled cross-over proof-of-concept study. Br J Anaesth . 2013;110(6):1010–6. http://dx.doi.org/10.1093/bja/aes578	No control group present for comparison.
13	Perelló M, Artés D, Pascuets C, Esteban E, Ey Batlle AM. Prolonged perioperative low-dose ketamine does not improve short and long-term outcomes after pediatric idiopathic scoliosis surgery. Spine (Phila Pa 1976). 2017;42(5):E304–12. http://dx.doi.org/10.1097/BRS.0000000000001772	No outcomes to pain relief
14	Pickering G, Pereira B, Morel V, Corriger A, Giron F, Marcaillou F, et al. Ketamine and magnesium for refractory neuropathic pain. Anesthesiology. 2020;133(1):154–64. http://dx.doi.org/10.1097/ALN.0000000000003345	Same patients in different times
15	Pöyhiä R, Vainio A. Topically administered ketamine reduces capsaicin-evoked mechanical hyperalgesia. Clin J Pain. 2006;22(1):32–6. http://dx.doi.org/10.1097/01.ajp.0000149800.39240.95	Same patients in different times
16	Schwartzman RJ, Alexander GM, Grothusen JR, Paylor T, Reichenberger E, Perreault M. Outpatient intravenous ketamine for the treatment of complex regional pain syndrome: a double-blind placebo controlled study. Pain. 2009;147(1–3):107–15. http://dx.doi.org/10.1016/j.pain.2009.08.015	Same patients in different times
17	Sen H, Sizlan A, Yanarates O, Emirkadi H, Ozkan S, Dagli G, et al. A comparison of gabapentin and ketamine in acute and chronic pain after hysterectomy. Anesth Analg. 2009;109(5):1645–50. http://dx.doi.org/10.1213/ANE.0b013e3181b65ea0	No control group present for comparison.
18	Timm C, Linstedt U, Weiss T, Zenz M, Maier C. Sympathomimetische Effekte auch bei niedriger Dosierung von Esketamin: Einfluss von Propofoldosierungen. Anaesthesist. 2008;57(4):338–46. http://dx.doi.org/10.1007/s00101-008-1331-0	No control group present for comparison.
19	Vaid P, Green T, Shinkaruk K, King-Shier K. Low-dose ketamine infusions for highly opioid-tolerant adults following spinal surgery: A retrospective before-and-after study. Pain Manag Nurs. 2016;17(2):150–8. http://dx.doi.org/10.1016/j.pmn.2016.01.003	Data isn't available
20	Baad-Hansen L, Juhl GI, Jensen TS, Brandsborg B, Svensson P. Differential effect of intravenous S-ketamine and fentanyl on atypical odontalgia and capsaicin-evoked pain. Pain. 2007;129(1–2):46–54. http://dx.doi.org/10.1016/j.pain.2006.09.032	No control group present for comparison.
21	Backonja M, Arndt G, Gombar KA, Check B, Zimmermann M. Response of chronic neuropathic pain syndromes to ketamine: a preliminary study. Pain. 1994;56(1):51–7. http://dx.doi.org/10.1016/0304-3959(94)90149-X	Same patients in different times.
22	Chumbley GM, Thompson L, Swatman JE, Urch C. Ketamine infusion for 96 hr after thoracotomy: Effects on acute and persistent pain. Eur J Pain. 2019;00:1–6. https://doi.org/10.1002/ejp.1366	Data isn't available.

23	Dahan A, Olofsen E, Sigtermans M, Noppers I, Niesters M, Aarts L, et al. Population pharmacokinetic-pharmacodynamic modeling of ketamine-induced pain relief of chronic pain. Eur J Pain. 2011;15(3):258–67. http://dx.doi.org/10.1016/j.ejpain.2010.06.016	Data isn't available.
24	Delage N, Morel V, Picard P, Marcaillou F, Pereira B, Pickering G. Effect of ketamine combined with magnesium sulfate in neuropathic pain patients (KETAPAIN): study protocol for a randomized controlled trial. Trials. 2017 Nov 3;18.https://www.ncbi.nlm.nih.gov/pmc/articles/PMC5670712/	No control group present for comparison.
25	Eide PK, Jørum E, Stubhaug A, Bremnes J, Breivik H. Relief of post-herpetic neuralgia with the Symbol receptor antagonist ketamine: A double-blind, cross-over comparison with morphine and placebo. Pain. 1994;58(3):347–54. http://dx.doi.org/10.1016/0304-3959(94)90129-5	No control group present for comparison.
26	Eide PK, Stubhaug A. Relief of glossopharyngeal neuralgia by ketamine-induced /V-methyl-aspartate receptor blockade. Neurosurgery. 1997;41(2):505–8. http://dx.doi.org/10.1097/00006123-199708000-00043	Compilation of studies.
27	Fallon MT, Wilcock A, Kelly CA, Paul J, Lewsley L-A, Norrie J, et al. Oral ketamine vs placebo in patients with cancer-related neuropathic pain: A randomized clinical trial. JAMA Oncol. 2018;4(6):870–2. http://dx.doi.org/10.1001/jamaoncol.2018.0131	No outcomes to pain relief.
28	Felsby S, Nielsen J, Arendt-Nielsen L, Jensen TS. NMDA receptor blockade in chronic neuropathic pain: a comparison of ketamine and magnesium chloride. Pain. 1996;64(2):283–91. http://dx.doi.org/10.1016/0304-3959(95)00113-1	Same patients at different times.
29	Gottrup H, Bach FW, Juhl G, Jensen TS. Differential effect of ketamine and lidocaine on spontaneous and mechanical evoked pain in patients with nerve injury pain. Anesthesiology. 2006;104(3):527–36. http://dx.doi.org/10.1097/00000542-200603000-00021	Same patients at different times.
30	Haines DR, Gaines SP. N of 1 randomised controlled trials of oral ketamine in patients with chronic pain. Pain. 1999;83(2):283–7. http://dx.doi.org/10.1016/s0304-3959(99)00117-7	Same patients at different times.
31	Huge V, Lauchart M, Magerl W, Schelling G, Beyer A, Thieme D, et al. Effects of low-dose intranasal (S)-ketamine in patients with neuropathic pain. Eur J Pain. 2010;14(4):387–94. http://dx.doi.org/10.1016/j.ejpain.2009.08.002	No control group present for comparison.
32	Jørum E, Warncke T, Stubhaug A. Cold allodynia and hyperalgesia in neuropathic pain: the effect of N-methyl-D-aspartate (NMDA) receptor antagonist ketamine--a double-blind, cross-over comparison with alfentanil and placebo. Pain. 2003;101(3):229–35. http://dx.doi.org/10.1016/S0304-3959(02)00122-7	No control group present for comparison.
33	Kannan TR, Saxena A, Bhatnagar S, Barry A. Oral ketamine as an adjuvant to oral morphine for neuropathic pain in cancer patients. J Pain Symptom Manage. 2002;23(1):60–5. http://dx.doi.org/10.1016/s0885-3924(01)00373-6	No control group present for comparison.
34	Kim YH, Lee PB, Oh TK. Is magnesium sulfate effective for pain in chronic postherpetic neuralgia patients comparing with ketamine infusion therapy? J Clin Anesth. 2015;27(4):296–300. http://dx.doi.org/10.1016/j.jclinane.2015.02.006	No control group present for comparison.

35	Kvarnström A, Karlsten R, Quiding H, Emanuelsson B-M, Gordh T. The effectiveness of intravenous ketamine and lidocaine on peripheral neuropathic pain: Analgesic effect on neuropathic pain. Acta Anaesthesiol Scand. 2003;47(7):868–77. http://dx.doi.org/10.1034/j.1399-6576.2003.00187.x	No control group present for comparison.
36	Kvarnström A, Karlsten R, Quiding H, Gordh T. The analgesic effect of intravenous ketamine and lidocaine on pain after spinal cord injury. Acta Anaesthesiol Scand. 2004;48(4):498–506. http://dx.doi.org/10.1111/j.1399-6576.2003.00330.x	No control group present for comparison.
37	Leung A, Wallace MS, Ridgeway B, Yaksh T. Concentration–effect relationship of intravenous alfentanil and ketamine on peripheral neurosensory thresholds, allodynia and hyperalgesia of neuropathic pain. Pain. 2001;91(1):177–87. http://dx.doi.org/10.1016/s0304-3959(00)00433-4	No control group present for comparison.
38	Lynch ME, Clark AJ, Sawynok J, Sullivan MJL. Topical 2% amitriptyline and 1% ketamine in neuropathic pain syndromes: a randomized, double-blind, placebo-controlled trial. Anesthesiology. 2005;103(1):140–6. http://dx.doi.org/10.1097/00000542-200507000-00021	Data isn't available
39	Max MB, Byas-Smith MG, Gracely RH, Bennett GJ. Intravenous infusion of the NMDA antagonist, ketamine, in chronic posttraumatic pain with allodynia: A double-blind comparison to alfentanil and placebo. Clin Neuropharmacol . 1995;18(4):360–8. http://dx.doi.org/10.1097/00002826-199508000-00008	Same patients in different times.
40	Mendola C, Cammarota G, Netto R, Cecci G, Pisterna A, Ferrante D, Casadio C, Della Corte F. S(+)-ketamine for control of perioperative pain and prevention of post thoracotomy pain syndrome: a randomized, double-blind study. Minerva Anestesiol. 2012 Jul;78(7):757-66. Epub 2012 Mar 22. PMID: 22441361.	Data isn't available.
41	Niesters M, Hoitsma E, Sarton E, Aarts L, Dahan A. Offset analgesia in neuropathic pain patients and effect of treatment with morphine and ketamine. Anesthesiology. 2011;115(5):1063–71. http://dx.doi.org/10.1097/ALN.0b013e31822fd03a	Same patients in different times
42	Nikolajsen L, Hansen CL, Nielsen J, Keller J, Arendt-Nielsen L, Jensen TS. The effect of ketamine on phantom pain: a central neuropathic disorder maintained by peripheral input. Pain.1996;67(1):69–77. http://dx.doi.org/10.1016/0304-3959(96)03080-1	No control group present.
43	Persson J, Hasselström J, Wiklund B, Heller A, Svensson JO, Gustafsson LL. The analgesic effect of racemic ketamine in patients with chronic ischemic pain due to lower extremity arteriosclerosis obliterans. Acta Anaesthesiol Scand. 1998;42(7):750–8. http://dx.doi.org/10.1111/j.1399-6576.1998.tb05317.x	No control group present.
44	Touil N, Pavlopoulou A, Barbier O, Libouton X, Lavand'homme P. Evaluation of intraoperative ketamine on the prevention of severe rebound pain upon cessation of peripheral nerve block: a prospective randomised, double-blind, placebo-controlled study. Br J Anaesth. 2022;128(4):734–41. http://dx.doi.org/10.1016/j.bja.2021.11.043	Data isn't available
45	Aveline C, Roux AL, Hetet HL, Gautier JF, Vautier P, Cognet F, et al. Pain and recovery after total knee arthroplasty: a 12-month follow-up after a prospective randomized study evaluating Nefopam and Ketamine for early rehabilitation. Clin J Pain. 2014;30(9):749–54. http://dx.doi.org/10.1097/AJP.0000000000000033	Same patients at different times.

Table S3: GRADE. Summary of Findings (SoF) and quality of evidence (GRADE) for Duloxetine in patients with neuropathic pain associated.

Certainty assessment							№ of patients		Effect		Quality of evidence (GRADE)	Importance
№ of studies	Study design	Risk of bias	Inconsistency	Indirectness	Imprecision	Other considerations	Duloxetine	Control group	Relative (95% CI)	SMD (95% CI)		
**NRS LDK first month**												
**3**	RCT	Not Serious	Serious	Not serious	Serious	None	84	82	-	-0.44 (-0.64 to -0.24)	⊕⊕○○ Low	CRITICAL
NRS LDK third month												
2	RCT	Not Serious	Very serious	Not serious	Serious	None	112	116	-	-0.46 (-0.61 to -0.32)	⊕○○○ Very low	CRITICAL
VAS LDK first month												
2	RCT	Not serious	Very serious	Not serious	Serious	None	84	82	-	55(-1.14 to 0.05)	⊕⊕○○ Low	CRITICAL

SMD: Standard Mean Difference; **RCT:** Randomized clinical trial; **Quality of evidence:** High: The research provides a very good indication of the likely effect. The probability that the effect is different is low; Moderate: The research provides a good indication of the likely effect. The probability that the effect is substantially different is moderate; Low: The research gives some indication of the probable effect. However, the probability that the effect is substantially different is high; Very low: The research does not provide a reliable estimate of the probable effect. The probability that the effect is substantially different is very high. **Downgrading:** GRADE approach has four reasons for possible rate down the quality of evidence. Begins with the study designs (trials or observational studies), secondly downgrading the evidence one level: (1) for study limitation if the majority of studies (>50%) was rated as high risk of bias; (2) for inconsistency, if heterogeneity was greater than the accepted low level (I^2 >40%); (3) for indirectness, directness was undoubled; (4) for imprecision, if meta-analysis had a small sample size (n <400) or confidence interval very wide.
